# Transformation Kinetics of Burnt Lime in Freshwater and Sea Water

**DOI:** 10.3390/ma13214926

**Published:** 2020-11-02

**Authors:** Harald Justnes, Carlos Escudero-Oñate, Øyvind Aaberg Garmo, Martin Mengede

**Affiliations:** 1 SINTEF Community, Stiftelsen for Industriell og Teknisk Forskning (SINTEF), Høgskoleringen 7b, 7034 Trondheim, Norway; 2 Faculty of Natural Sciences, Norwegian University of Science and Technology (NTNU), Sem Sælandsvei 12, 7034 Trondheim, Norway; 3 Norwegian Institute for Water Research (NIVA), Gaustadalléen 21, 0349 Oslo, Norway; Carlos.Escudero@niva.no (C.E.-O.); oyvind.garmo@niva.no (Ø.A.G.); 4 Franzefoss Minerals AS, Olav Ingstads vei 5, 1309 Rud, Norway; martin.mengede@kalk.no

**Keywords:** calcium oxide, calcium hydroxide, kinetics, lime, magnesium hydroxide, sea water

## Abstract

Calcium oxide (CaO), also known as burnt lime, is being considered as a possible treatment to reduce the negative impact of sea urchins on tare forests in northern coastal waters and blue-green algal blooms in the surrounding of fish-farms. In this respect, the reaction kinetics of burnt lime in contact with sea water has been elucidated and compared to its behaviour in fresh water. In the first minutes of contact between burnt lime and water, it “slaked” as CaO reacted with water to yield calcium hydroxide (Ca(OH)_2_). Subsequently, calcium hydroxide reacted with magnesium, sulphate and carbonate from the sea water to yield magnesium hydroxide (Mg(OH)_2_), calcium sulphate dihydrate (gypsum, CaSO_4_·2H_2_O) and calcium carbonate (CaCO_3_), respectively. In a closed system of 1% CaO in natural sea water (where the supply of sulphate, magnesium and carbonate is limited), more than 90% reacted within the first 5 h. It is foreseen that in an open system, like a marine fjord, it will react even faster. The pH 8 of sea water close to the CaO particle surface will immediately increase to a theoretical value of about 12.5 but will, in an open system with large excess of sea water, rapidly fall back to pH 10.5 being equilibrium pH of magnesium hydroxide. This is further reduced to <9 due to the common ion effect of dissolved magnesium in sea water and then be diluted to the sea water background pH, about 8. Field test dosing CaO particles to sea water showed that the pH of water between the particles stayed around 8.

## 1. Introduction

### 1.1. Applications of Burnt Lime in Sea Water

To combat the negative impact of sea urchins on tare forests in northern coastal waters and blue-green algal blooms in the surrounding of fish-farms (monoculture), Brooks et al. [1] and Strand et al. [2] has investigated addition of burnt lime (calcium oxide, CaO) to sea water as a remedial action. The treatment involves a rapid suspension of burnt lime in sea water and immediate discharge into the marine environment. Hence, it becomes of paramount importance to know how CaO particles behaves in contact with sea water and how this substance modifies the water chemistry.

The aim of this treatment is to increase tare growth and population density. This would provide a habitat for many species and will increase the biodiversity of the marine environment.

On a longer term one can imagine harvesting of high-quality sea urchins for the food market as well as a preparedness plan to treat blue-green algal blooms for the same market. In a wider perspective lime can be used to capture CO_2_ in sea water as an environmental remediation [3,4]. However, designing an efficient treatment strategy to tackle the variable conditions expected in the sea requires increased understanding of the reaction kinetics of CaO in sea water, and this paper is an effort to elucidate this in comparison to fresh water as a novelty.

The burnt lime treatment is usually performed by spraying a suspension of CaO particles directly at the surface of the sea and all these particles will interfere with each other in their microenvironment until the gradual dilution is so great that they can be considered as single particles. For instance, the treatment in practice consists of pumping a slurry of 100 kg burnt lime (0.2–0.8 mm diameter) dispersed in 600 litres of sea water onto the surface. This provides an initial CaO concentration in the slurry of 167 g/L, that is taken as a starting point. Assuming that the average particle diameter is 0.5 mm and that they are spherical, the volume of the particle will be $\frac{4}{3}{\pi r}^{3}$ or 0.0654 mm^3^. Light-burnt lime (burnt at 1060 °C) has a typical particle density [5] of ρ_p_ = 2 g/cm^3^. The particle is porous since CaO has solid density ρ_s_ = 3.35 g/cm^3^; porosity = (1 − ρ_p_/ρ_s_) × 100 vol% = 40 vol%, which is also what Wuhrer [6] found. This gives for a 167 g/L slurry an outer volume of light-burnt lime of 83.5 cm^3^ or in other words about 1275 particles per mL. These particles will however immediately react with water as discussed in the next Section about reaction with fresh water. The reaction with fresh water is evaluated first in order to illustrate the difference to sea water in the Section thereafter.

### 1.2. Chemistry of Burnt Lime (CaO) in Fresh Water

Burnt lime reacts violently with water to produce calcium hydroxide (so called slaked lime) according to the reaction below:

CaO + H_2_O = Ca(OH)_2_ (1)

The reaction in Equation (1) releases a heat of Q = 1160 J/g CaO. The heat capacity of water is c = 4.18 kJ/kg·K. The increase in temperature of a mass (m) of water due to the reaction in Equation (1) can be calculated by the formula in Equation (2);

ΔT = Q/c × m(2)

Using the same concentration of particles as in the preceding Section (167 g/L) and a density of water of 1000 g/L, one find that the water will be heated ΔT = 167 g × 1.160 kJ/g/(4.18 kJ/kg·K × 1 kg) = 46 K = 46 °C. This temperature increase is for a closed system (adiabatic, without heat loss) and 100% hydration of the burnt lime particles and will be proportional less for decreasing fractions of reaction. The temperature increase as a function of burnt lime dosage and fraction of hydration is given in Table 1 as an illustration.

**Figure 1:**
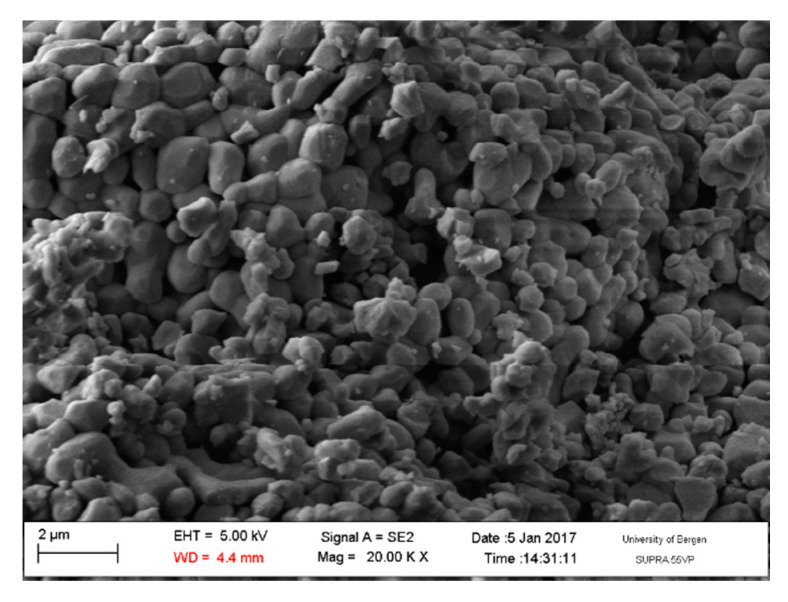
An electron microscopy image of the pore walls of an unhydrated light-burnt lime particle [7].

A light-burnt lime particle is not massive, but porous as shown in Figure 1 [7].

When the lime particle meets water, this will be sucked into the pores and react immediately to calcium hydroxide while the pore water temperature will increase rapidly to above the boiling point since there is a small amount of water relative to the CaO of the pore wall. If the precipitated calcium hydroxide has blocked the pore (Equation (1) doubles the volume of solid matter) the particle will disintegrate due to the thermal expansion of the remaining water and/or its vapor pressure. The reaction can be described as chaotic and will not be less chaotic when components from sea water interfere as described in next Section. Calcium hydroxide has limited solubility and the solubility actually decreases with increasing temperature as shown in Equation (3) (calculated from tabular data [8]), an anomaly compared to most other compounds. The reaction rate of CaO, on the other hand, increases with increasing temperature and the degree of reaction for a light-burnt lime particle may reach 80% after 1 h under isothermal conditions, but after <5 min under adiabatic conditions (time to reach 60 °C according to EN459-2) depending on the fineness of the burnt lime.

Solubility of Ca(OH)_2_ (g/L) = −0.0117 × T (°C) + 1.924 with r^2^ = 0.9954 (3)

In order to calculate the pH of burnt lime slaked in distilled water, one can use the solubility of 1.63 g/L at 25 °C (from Equation (3)) knowing that calcium hydroxide dissociates as written in Equation (4);

Ca(OH)_2_ (s) = Ca^2+^ + 2 OH^−^ with solubility product K_sp_ = [Ca^2+^] × [OH^−^]^2^ (4)

where the concentrations of ions in brackets are expressed in mol/L. According to Equation (4) the concentration of hydroxyl ions is twice that of the dissolved calcium hydroxide;

[OH^−^] = 2 × [Ca^2+^] = 2 × [Ca(OH)_2_] (5)

since M_w_ (Ca(OH)_2_) = 74.08 g/mol, [OH^−^] = 2 × 1.63 g/L/74.08 g/mol = 0.04405 mol/L

The relation between [OH^−^] and pH at 25 °C is;

pH = −log [H^+^] = 14.00 + log [OH^−^] (6)

since

H_2_O ↔ H^+^ + OH^−^ with dissociation constant K_d_ = [H^+^] × [OH^−^] = 1.0 × 10^−14^ at 25 °C (7)

pH = −log [H^+^] = 7 for neutral water since [H^+^] = [OH^−^] and [H^+^] = √(1.0 × 10^−14^).

[OH^−^] = 0.04405 M for dissolved calcium hydroxide gives then pH = 12.6 for a solubility of 1.63 g/L at 25 °C.

pH will be a bit lower than this as dissolved calcium hydroxide is not completely dissociated and species like CaOH^+^ exists;

(8) $${\mathrm{Ca}}^{2+}\;+\;{\mathrm{OH}}^{-}\;↔\;\mathrm{Ca}(\mathrm{OH}{)}^{+}\;\mathrm{with}\;\mathrm{solubility}\;\mathrm{product}\;K=\;\frac{\left[\mathrm{Ca}{\left(\mathrm{OH}\right)}^{+}\right]}{\left[{\mathrm{Ca}}^{2+}\right]\;\times \;\left[{\mathrm{OH}}^{-}\right]}$$

The geochemical data program GEMS (gems.web.psi.ch) [9,10] with PSI-GEMS thermodynamic data base for aqueous species and solids [11] minimizes Gibbs free energy and gives the following composition of a saturated solution of calcium hydroxide at 25 °C:

[Ca^+2^] = 0.016370 M or 0.65611 g/L
[CaOH^+^] = 0.004265 M or 0.24345 g/L
[OH^−^] = 0.037006 M or 0.62910 g/L
pH = 12.5

This gives an amount of dissolved calcium hydroxide of 1.53 g/L instead of the 1.63 g/L obtained through Equation (3) from [8], which explains the difference in calculated pH together with the presence of the species CaOH^+^. Due to this species the pH as measured by a pH-meter will be lower (12.5) than the corresponding amount of dissolved calcium hydroxide; pH = 12.6 for 1.63 g/L dissolved Ca(OH)_2_, which would have been the result if the solution was titrated rather than measured by a pH-meter. The proceeding evaluations will nevertheless be based on the solubility from Equation (3) (1.63 g/L resulting in pH = 12.6) and ignore the presence of species like CaOH^+^ since it will react with the different species in sea water as if it was separated as Ca^2+^ and OH^−^.

**Figure 2:**
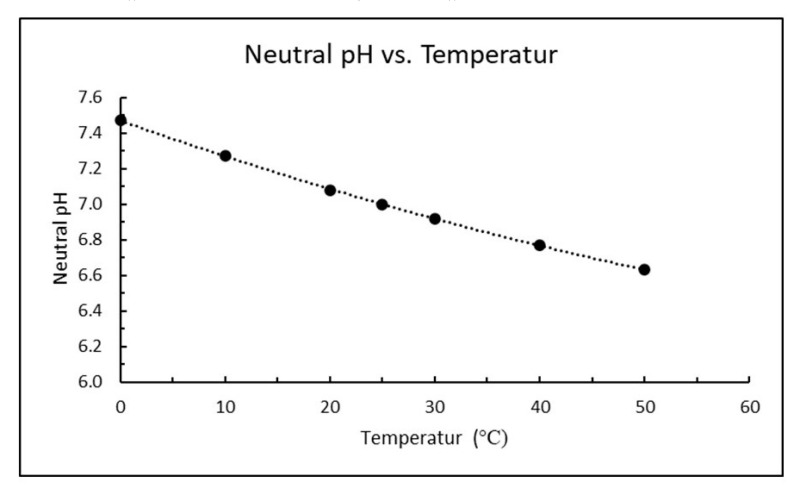
pH for neutral water as a function of temperature (t) in the range 0–50 °C plotted from tabular data [12]. The regression line follows formula neutral pH = 8 × 10^−5^ × t^2^ − 0.0208 × t + 7.4692 with regression factor r^2^ = 0.9999.

Another complicating factor for correct pH is that the self-dissociation of water in Equation (7) increases with increasing temperature as temperature is just a measure of molecular movement. The pH reference value of 14 will then increase with decreasing temperature, or pH of neutral water will decrease with increasing temperature as plotted in Figure 2. Neutral pH at 5 and 15 °C would be 7.4 and 7.2, respectively, which corresponds to reference values of 14.8 and 14.4 (rather than 14.0 in Equation (7)) unless the pH-meter is correcting for temperature.

The pH in a suspension of calcium hydroxide will further increase with decreasing temperature since the solubility of calcium hydroxide increases as shown by Equation (3).

Consider distilled water equilibrating with natural air containing 400 ppm or 0.04 vol% carbon dioxide. The CO_2_ can partly be dissolved as the molecule or react with water to hydrogen carbonate (so called carbonic acid):

(9) $$\begin{array}{c} {\mathrm{CO}}_{2}\;+\;H_{2}O\;↔\;H_{2}{\mathrm{CO}}_{3}\\ \mathrm{with}\;\mathrm{hydration}\;\mathrm{product}\;K_{h}=\;\frac{\left[H_{2}{\mathrm{CO}}_{3}\right]}{\left[{\mathrm{CO}}_{2}\;\left(\mathrm{aq}\right)\right]}=1.7\;\times \;10^{-3}\;\mathrm{at}\;25\;{}^{\circ}C \end{array}$$

The main component of dissolved CO_2_ will be molecular CO_2_ (aq), since K_h_ is so small that the concentration of H_2_CO_3_ would be negligible and do not influence pH (i.e., log [H^+^]). The total solubility of CO_2_ at 100 kPa CO_2_ pressure (about 1 atm) and 25 °C is 1.45 g/L.

Hydrogen carbonate dissociates according to 2 sequential steps:

(10) $$\begin{array}{c} H_{2}{\mathrm{CO}}_{3}\;↔\;H^{+}\;+\;{\mathrm{HCO}}_{3}^{-}\\ \mathrm{with}\;\mathrm{dissociation}\;\mathrm{constant}\;K_{a1}=\;\frac{\left[{\mathrm{HCO}}_{3}{}^{-}\right]\;\times \;\left[H^{+}\right]}{\left[H_{2}{\mathrm{CO}}_{3}\right]}=2.5\;\times \;10^{-4}\;\mathrm{at}\;25\;{}^{\circ}C \end{array}$$

(11) $$\begin{array}{c} {\mathrm{HCO}}_{3}^{-}\;↔\;H^{+}\;+\;{\mathrm{CO}}_{3}^{2-}\\ \mathrm{with}\;\mathrm{dissociation}\;\mathrm{constant}\;K_{a2}=\;\frac{\left[{\mathrm{CO}}_{3}{}^{2-}\right]\;\times \;\left[H^{+}\right]}{\left[{\mathrm{HCO}}_{3}{}^{-}\right]}=4.69\;\times \;10^{-11}\;\mathrm{at}\;25\;{}^{\circ}C \end{array}$$

**Figure 3:**
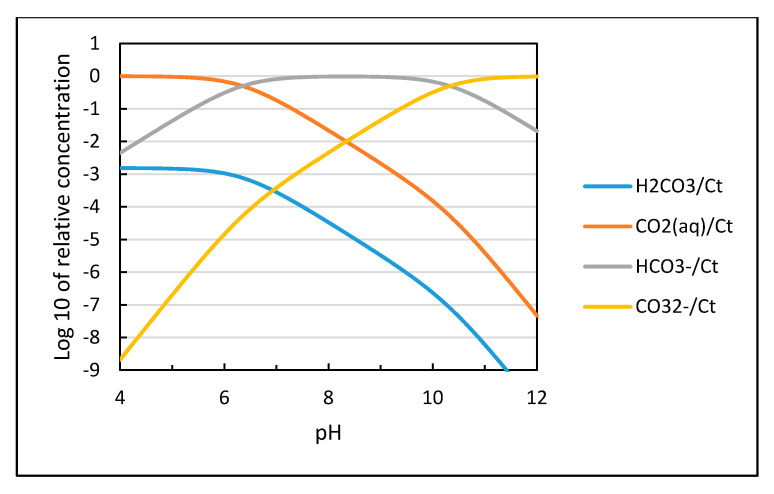
Distribution of solute species versus pH for a closed aqueous carbonate system at 25 °C and zero ionic strength plotted based on their equilibrium constants [13].

**Figure 4:**
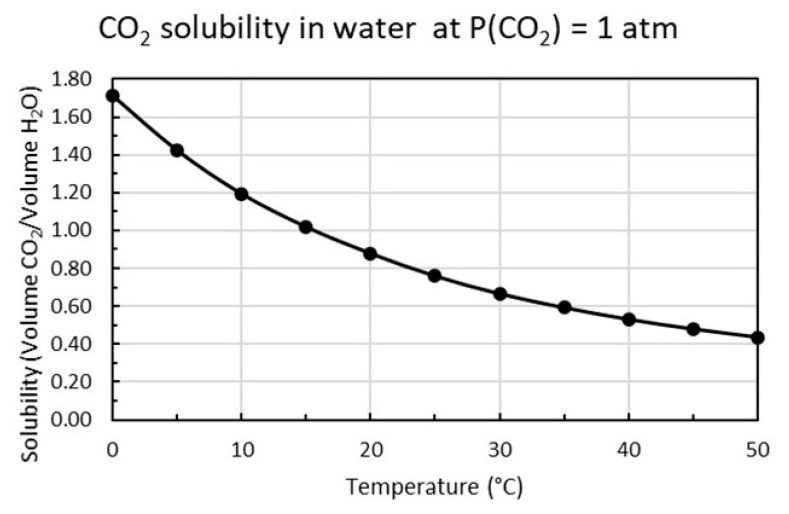
Dissolved CO_2_ versus temperature (°C) for carbon dioxide pressure P (CO_2_) = 1 atm plotted from tabular data [14].

The distribution of the different species for CO_2_ dissolved in water as a function of pH is shown in Figure 3 based on calculations using the equilibrium constants in Equations (9)–(11). The solubility of CO_2_ is proportional to the partial pressure of carbon dioxide as shown in Equation (12) and increases with decreasing temperature as plotted in Figure 4.

(12) $$\left[{\mathrm{CO}}_{2}]\;=\;k\;\times \;P({\mathrm{CO}}_{2}\right)$$

For two different pressures Equation (12) can be reformulated to

(13) $$k=\;\frac{{\left[{\mathrm{CO}}_{2}\right]}_{1}}{P_{1}\left({\mathrm{CO}}_{2}\right)}=\;\frac{{\left[{\mathrm{CO}}_{2}\right]}_{2}}{P_{2}\left({\mathrm{CO}}_{2}\right)}\;\mathrm{and}\;\mathrm{for}\;{\left[{\mathrm{CO}}_{2}\right]}_{2}=\;\frac{P_{2}\left({\mathrm{CO}}_{2}\right)}{P_{1}\left({\mathrm{CO}}_{2}\right)}\times {\left[{\mathrm{CO}}_{2}\right]}_{1}{}_{}$$

Figure 4 shows that at 15 °C and P_1_(CO_2_) = 1 atm the concentration of carbon dioxide is [CO_2_]_1_ = 2 g/L or 45.45 mM. If k is constant over the whole pressure range, the solubility at P_2_(CO_2_) = 0.04 atm will be [CO_2_]_2_ = 0.08 g/L or 1.82 mM.

When excess calcium hydroxide with pH = 12.6 interact with fresh water with dissolved CO_2_ the equilibria in Equations (9)–(11) will rapidly be displaced to the right and calcium carbonate will precipitate in an amount corresponding to the amount of dissolved CO_2_ regardless of species:

Ca(OH)_2_ + CO_2_ (aq) = CaCO_3_ (s) + H_2_O (14)

If the amount of calcium hydroxide is far above saturation the pH will be unchanged at the same time as calcium carbonate is precipitating since the solubility of calcium carbonate is very small (≈ 0.014 g/L). The calcium concentration for saturated calcium hydroxide is about 22 mM [Ca^2+^] and much higher than for saturated calcium carbonate having 0.14 mM [Ca^2+^], a difference of about 150 times. This means that for thinning of an exactly saturated calcium hydroxide solution, the pH is controlled by dissolved CO_2_:

pH = 14.00 + log {2·([Ca(OH)_2_] − [CO_2_])} (15)

In natural fresh water with dissolved alkali carbonate (CO_3_^2−^), alkali hydrogen carbonate (HCO_3_^−^) and magnesium (Mg^2+^) dissolved calcium hydroxide will react as follows:

Ca(OH)_2_ + HCO_3_^−^ = CaCO_3_ (s) + H_2_O + OH^−^ (16)

Ca(OH)_2_ + CO_3_^2−^ = CaCO_3_ (s) + 2 OH^−^ (17)

Ca(OH)_2_ + Mg^2+^ = Mg(OH)_2_ (s) + Ca^2+^ (18)

The reactions in Equations (16)–(18) leads to precipitates, since the solubilities at 25 °C for calcite, CaCO_3_, is 0.014 g/L or 0.14 mM, and for brucite, Mg(OH)_2_, 0.009 g/L or 0.15 mM. Calcite and brucite are considerably less soluble than portlandite (mineral name for calcium hydroxide), Ca(OH)_2_, with 1.63 g/L or 22 mM (a factor of about 150 in molarity).

For fresh water saturated with respect to calcium hydroxide, the reactions in Equations (16) and (17) will lead to a pH increase since soluble alkali hydroxides will form if the water contains alkali carbonates or alkali hydrogen carbonates. The reaction in Equation (18) will lead to a weak reduction in pH since it will increase the concentration of calcium ions that in turn will suppress the solubility of calcium hydroxide due to the common ion effect according to Equation (4).

For fresh water undersaturated with respect to calcium hydroxide, reaction 16 will reduce pH, reaction 17 not lead to any change and reaction 18 reduce pH if brucite is precipitated.

On long term, all calcium hydroxide in fresh water will be converted to calcium carbonate as there is an unlimited supply of CO_2_ relative to calcium hydroxide while the rate is dependent on the concentration of CO_2_. Pure fresh water without dissolved CO_2_ in equilibrium with calcite (CaCO_3_) has pH 9.9 [15] but will be somewhat lower if there is dissolved CO_2_ present as well. Based on the calcite solubility of 0.14 mM at 25 °C the pH should be about 10, but there is some uncertainty in the solubility.

### 1.3. Chemistry of Burnt Lime (CaO) in Sea Water

Atlantic sea water has typical composition listed in Table 2 and the sum of components gives a mass of 3.513%, which is a typical salinity. The sum of positive charge is 0.623 M, while the sum of negative charge is 0.622 M, confirming electroneutrality and that all major ions are accounted for. Since sea water consists of a mixture of dissolved salts, one cation does not belong to a specific anion to make a compound.

The greatest difference between sea water and fresh water is the content of Na^+^ and Cl^−^. Sodium chloride is not expected to form compounds with calcium hydroxide, but according to Duschesne and Reardon [16] the solubility of Ca(OH)_2_ increases with increasing concentration of NaCl due to ion pair formations in their calculations, which were verified experimentally by Johnston and Grove [17] and by Yeatts and Marshall [18]. If a simplified version of sea water is 0.5 M NaCl, the solubility of Ca(OH)_2_ will increase from 22 to 28 mM. On the other hand, a number of other ions in sea water will interact with Ca(OH)_2_ and disturb this picture.

The second biggest difference is the content of sulphate and much higher concentration of magnesium. The magnesium will react as described in Equation (18), while the sulphate ions will form gypsum:

Ca(OH)_2_ + SO_4_^2−^ +2 H_2_O = CaSO_4_·2H_2_O + 2 OH^−^ (19)

The magnesium reaction (Equation (18)) release calcium ions that suppress hydroxide concentration and gypsum formation (Equation (19)) will release hydroxyl ions that potentially reduce the calcium concentration locally relative to solid calcium hydroxide (Equation (4)), so the overall outcome is complex depending on concentration. According to Table 2 the molarity of magnesium is 0.0549 M in Atlantic sea water as compared to 0.0289 M for sulphate, so in sea water the magnesium reaction will dominate.

The potential compounds formed by interaction of calcium hydroxide and sea water as listed in Table 3 are predicted based on possible ion pairs and solubility data [19]. 

The magnesium compounds with higher molar solubility than brucite, Mg(OH)_2_, are not expected to form on its expense. An experimental program was then initiated to measure which compounds that are actually formed when burnt lime is added to sea water.

## 2. Materials and Methods

### 2.1. Materials

**Figure 5:**
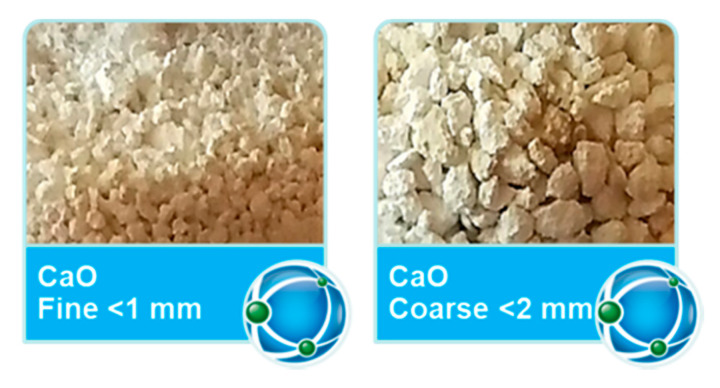
Physical appearance of fine CaO to the left and coarse CaO to the right.

**Figure 6:**
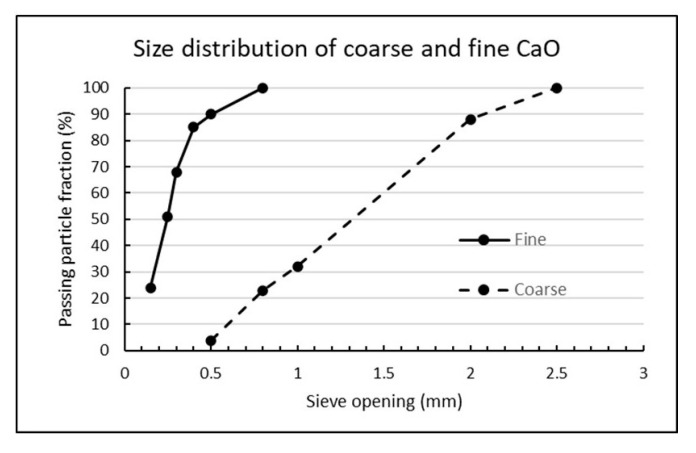
Sieving curves for fine and coarse CaO (burnt lime).

The burnt lime (CaO) used in all tests is of industrial grade from Miljøkalk AS, Verdal, Norway. There are two types denoted “Fine, burnt lime” and “Coarse, burnt lime” and the physical difference can be observed directly in Figure 5, while the sieving curves are shown in Figure 6. The sieving was performed in accordance with EN 933-1. Other properties of the burnt lime samples are given in Table 4.

### 2.2. Equipment

#### 2.2.1. Characterization Tools for the Solid Phase

Isothermal calorimetry of cement pastes was performed with a TAM (TA Instruments, New Castle, DE, USA) Air instrument set to 15 °C in closed glass vials. The glass vials were coupled to an external mixing device with 4 syringes with 1 mL capacity each for injection of either fresh or sea water. 70 mg of coarse or fine fraction of CaO was weighed into the glass vial, the syringes filled with 4 mL fresh or sea water and the whole arrangement closed in the isothermal calorimeter for thermal equilibration verified by zero heat flow. The stirring motor was the only part outside the calorimeter. Each experiment was repeated 3 times and the heat flow curves used was an average of 3. A dosage of 70 mg CaO/4 mL corresponds to 17.5 g/L.

Thermogravimetric analysis (TGA) was performed with a Mettler Toledo TGA/SDTA 851 (Greifensee, Switzerland). Samples were analysed with a heating rate of 10 °C/min between 40–900 °C. All measurements were performed in nitrogen atmosphere with a flow rate of 50 mL/min. The calcium hydroxide content in a hydrated CaO sample was calculated from the mass loss between ≈ 400–550 °C caused by its dehydration. The corresponding temperature range for thermal dehydration of magnesium hydroxide is 300–400 °C and the decarbonation of precipitated calcium carbonate for temperatures >600 °C. The exact boundaries were chosen from the peak in the DTG curve.

The mineralogy was detected using a Bruker D8-Advance with a generator of x-ray KRISTALLOFLEX K 760-80F (power: 3000 W, voltage: 20–60 KV and current: 5–80 mA) (Bruker D8-Advance, Billerica, MA, USA) with a tube of RX with copper anode was employed to record the diffractograms of the CaO in the kinetic experiments. All the measurements were taken at 25 °C from 10 to 70 2θ with a step size of 0.05° 2θ and 3 s time per step.

The morphology and the local chemical composition of the materials was explored in the time-course experiments using by Scanning Electron Microscopy (SEM) coupled to Energy Dispersive X-Ray Analysis (EDX). For these analyses a Hitachi S-3000 N Electron Microscope (Tokyo, Japan) coupled to an EDX Bruker Esprit 1.8 unit (Billerica, MA, USA) was employed.

#### 2.2.2. Analysis in the Liquid Phase

Fine CaO was added to natural sea water sampled from the Trondheim Fjord, city, Norway, at temperatures 5 or 15 °C in a surplus dosage of 10 g CaO/L during stirring. The experiments were performed in a container open to the atmosphere and the temperature of the surrounding environment was kept constant either by immersion in an ice-water bath (5 °C) or kept in a thermostatic room set at 15 °C. Before starting the experiments, the water was pre-adjusted to the required temperature by a thermostat.

The ion composition of the applied sea water is listed in Table 5. Samples of ≈ 400 μL was drawn from this suspension by a syringe at the times 0 (sea water before addition of CaO), 1, 3, 10, 15 and 30 min. Thereafter solution was drawn every hour until 8 h and a final sampling after 24 h. The sampled suspension was filtered through 0.45 μm syringe-filter and 100 μL of the filtrate was diluted to 10 mL using MilliQ water and analysed by Ion Chromatography (Dionex^®^, Sunnyvale, CA, USA) for F^−^, Cl^−^, Br^−^, SO_4_^2−^, CO_3_^2−^ (given as µS·min by the instrument), Na^+^, Mg^2+^, K^+^ and Ca^2+^. The analytical system was based on a double channel so both cations and anions were analysed under the same run. A Dionex IonPac CS16 column was used for detection of cations while a column Dionex AS18 coupled with a column CG5A was used for measurement of anions. The pH was measured by a pH-meter in a GLP/GMP environment calibrated just before the experiments towards pH = 7.0 and a 10.012 standard, as well as corrected with respect to temperature.

#### 2.2.3. Exposure Assays of CaO

Fine CaO was dispersed into sea water in a dosage of 5 g CaO per 500 mL sea water at 5 °C under slow stirring, and the suspension was kept under constant stirring between each sampling. Solids from the suspensions was rapid filtered off using filter paper on Büchner funnel attached to a vacuum pump followed by washing with 99% ethanol to halt the reaction. The solids were then added to a plastic tube with screw cap together with 15 mL ethanol and shaken for 30 s. This was followed by centrifugation at 1500 rpm and the liquid phase removed. Finally, the samples were freeze dried under vacuum for 24 h before sealed storage prior to further solid-phase analysis. The sampling was performed after 1, 5, 15 and 30 min, as well as after 1, 2, 5, 8, 24 h (1 day) and 2, 3, 4, 5, 6 and 7 days.

The solids produced in the time-course transformation experiments were investigated by SEM/EDX for topology and semi-quantitative local chemical composition. X-ray diffraction (XRD) was applied to follow qualitatively how the crystalline phases developed as a function of time, while TGA was used to determine the content of some phases quantitatively.

## 3. Results

### 3.1. Influence of Burnt Lime on Sea Water Composition as Function of Time

The salinity of the applied sea water in Table 5 is 3.448%, which is marginally lower than average Atlantic sea water given in Table 2 (3.513%). The sum of positive charge is 0.6149 mol/L while the sum of negative charge is 0.5824 mol/L. The lack of negative charge (Δ = 0.0325 mol/L) is attributed to carbonate and hydrogen carbonate not measured, as well as uncertainty in measurements.

**Figure 7:**
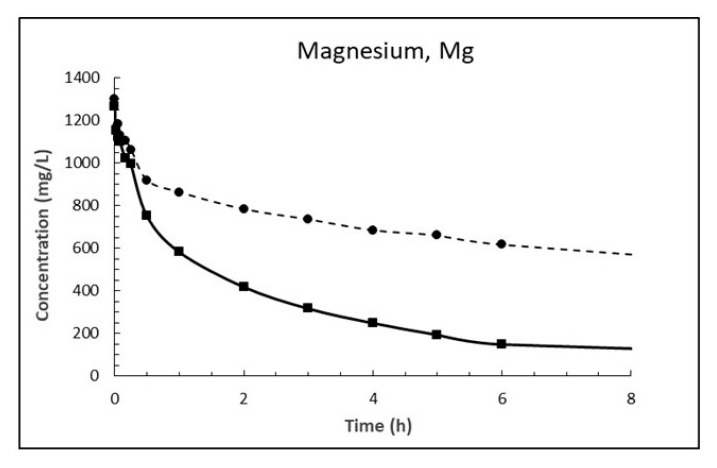
Concentration of magnesium in sea water at T = 5 °C (dashed line) and 15 °C (solid line) as function of time after adding 1% (*w*/*v*) fine CaO.

**Figure 8:**
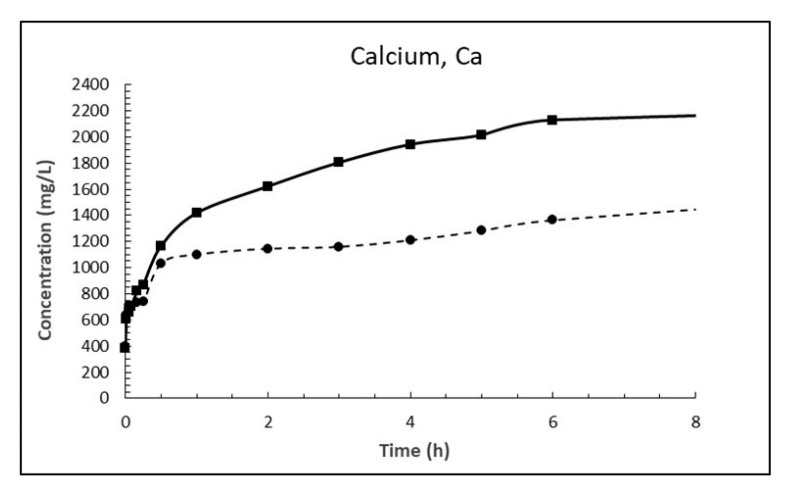
Concentration of calcium in sea water at T = 5 °C (dashed line) and 15 °C (solid line) as function of time after adding 1% (*w*/*v*) fine CaO.

**Figure 9:**
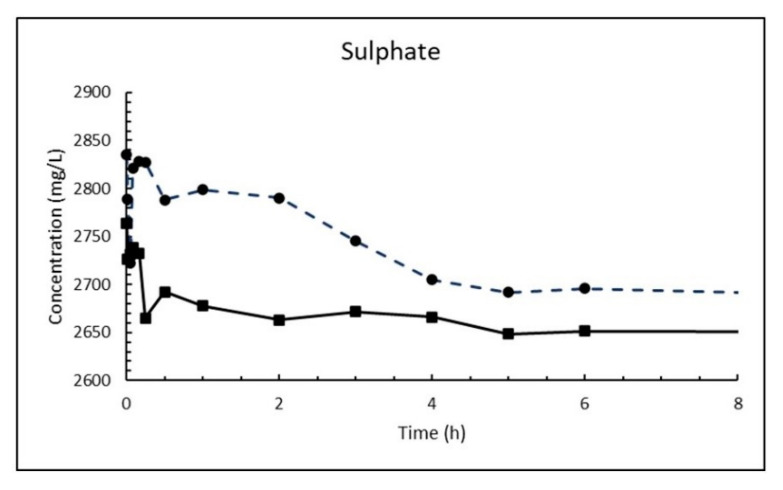
Concentration of sulphate in sea water at T = 5 °C (dashed line) and 15 °C (solid line) as function of time after adding 1% (*w*/*v*) fine CaO.

**Figure 10:**
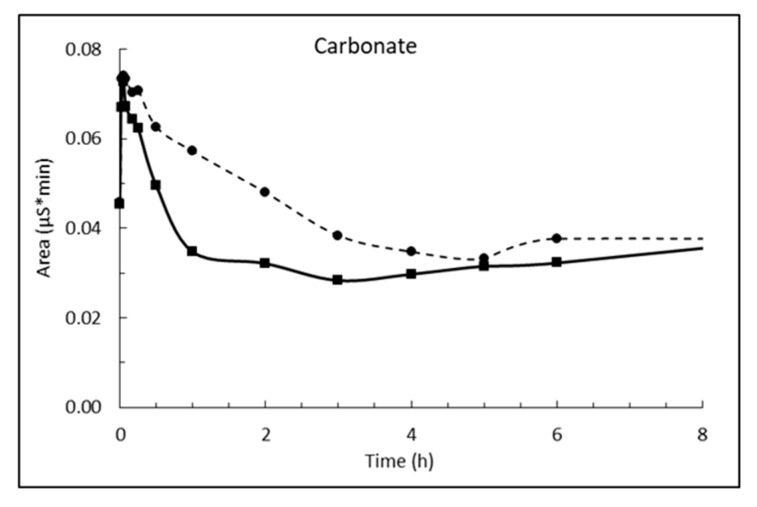
Relative concentration of carbonate in sea water at T = 5 °C (dashed line) and 15 °C (solid line) as function of time after adding 1% (*w*/*v*) fine CaO.

**Figure 11:**
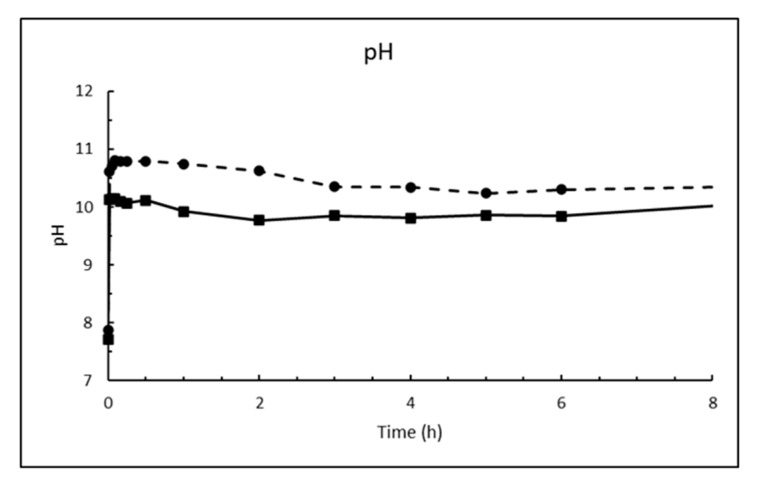
pH in sea water at T = 5 °C (dashed line) and 15 °C (solid line) as function of time after adding 1% (*w*/*v*) fine CaO.

The dosage of 10 g/L CaO in sea water corresponds to 13.2 g/L Ca(OH)_2_, which is about eight times higher than its solubility. The result is a white suspension often referred to as «milk of lime». The evolutions of magnesium (Mg^2+^), calcium (Ca^2+^), sulphate (SO_4_^2−^), carbonate (CO_3_^2−^) and pH in sea water as a function of time until 8 h after addition of burnt lime are plotted in Figure 7, Figure 8, Figure 9, Figure 10 and Figure 11, respectively. The ion concentrations after 24 h are presented in Table 6.

### 3.2. Change of Solids Composition as Function of Time for CaO in Contact with Sea Water

**Figure 12:**
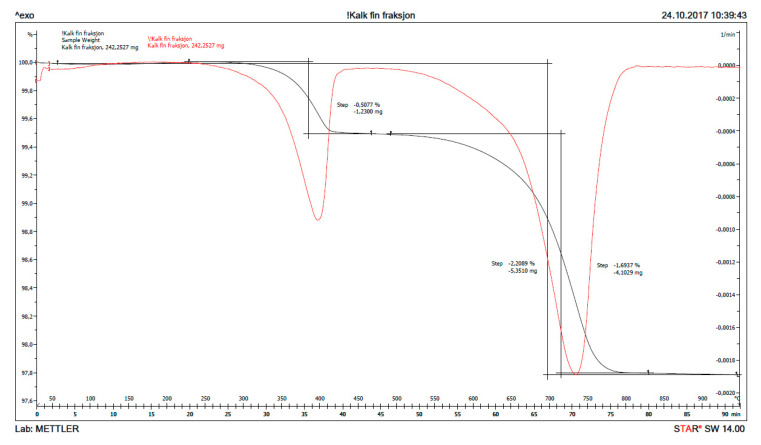
The thermogravimetry curves (TG) in black and its derivative (DTG) in red for fine CaO as received.

**Figure 13:**
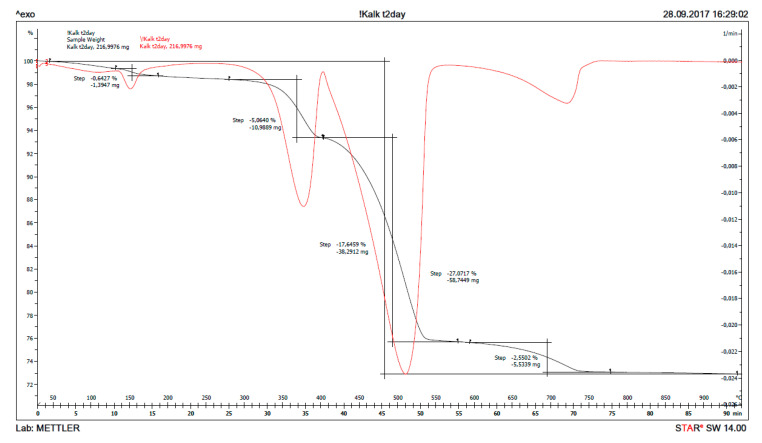
The thermogravimetry curves (TG) in black and its derivative (DTG) in red for the solids of 1% CaO dispersed in sea water for 2 days.

The result from thermogravimetry, TG (mass loss as function of temperature) is plotted in Figure 12 for fine, burnt lime (CaO) as received and in Figure 13 for the solids obtained after 1% CaO has been dispersed in sea water for two days. The signal with maximum for the derived curve (DTG) at ≈ 400 °C in Figure 12 is due to the decomposition of Ca(OH)_2_ to CaO and H_2_O, while the signal at ≈ 740 °C is caused by decomposition of CaCO_3_ to CaO and CO_2_. The signal just looks large in this accurate measurement due to the scale, but the bulk calculation from the curves only calculates to 2.09% Ca(OH)_2_ and 3.85% CaCO_3_ with the remainder being 94.06% CaO. The XRD of the same sample revealed largely signals from CaO with traces of Ca(OH)_2_ and CaCO_3_. It is difficult to avoid that a material as reactive as fine CaO will pick up some moisture and maybe carbon dioxide from air during handling.

**Figure 14:**
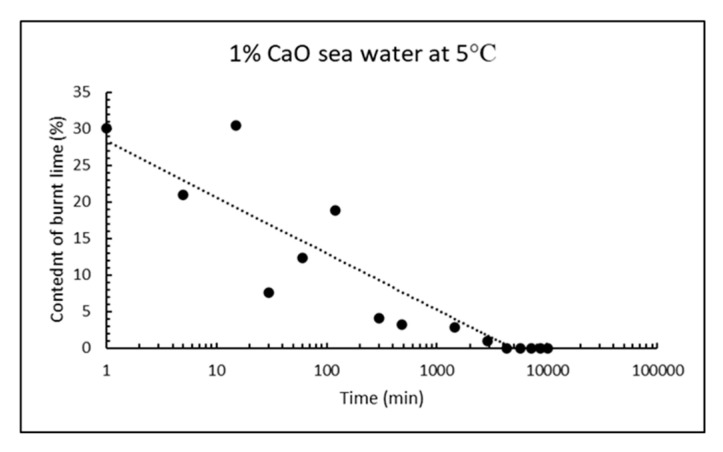
Remaining content of CaO as a function of time after 1% fine CaO have been mixed in sea water at 5 °C.

Similar calculations as for received CaO can be done when it has been reacting with sea water for some time, for instance from the curves after two days reaction in Figure 13. The signals with maxima at ≈ 140, 370, 510 and 730 °C are due to thermal decomposition of gypsum (loses two water molecules), magnesium hydroxide (loses one water molecule), calcium hydroxide (loses one water molecule) and calcium carbonate (loses one molecule carbon dioxide). From the TG/DTG data, the bulk composition of solids from reaction of CaO with sea water at any point in time can be calculated for the five phases: CaSO_4_⸳H_2_O (gypsum), Mg(OH)_2_ (brucite), Ca(OH)_2_ (portlandite), CaCO_3_ (sum of crystal modifications calcite, aragonite and vaterite), as well as remaining CaO (burnt lime) from the material mass weighed in, after subtracting the other five phases. The chemical reasoning in Section 1.3 postulate that only these phases will be present, and this is also confirmed by XRD after reacting 1% CaO with sea water for two days. The bulk composition of solids as a function of reaction time for 1% fine CaO in sea water is shown in Table 7 for the first day and in Table 8 for a week, while the content of remaining CaO is plotted in Figure 14. The deviation from 100% in Table 7 and Table 8 is due to adsorbed moisture. The results reveal that more than 97% CaO has reacted after one day and as much as 96% after just 5 h.

**Figure 15:**
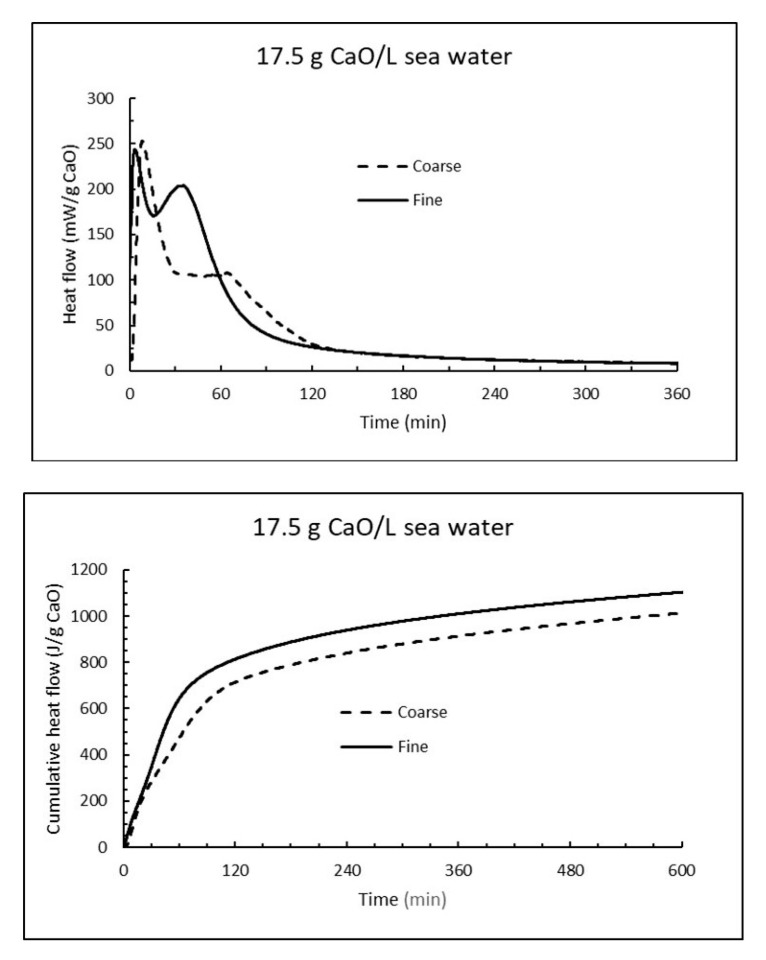
The heat flow (**top**) and cumulative heat (**bottom**) under isothermal conditions at 15 °C when 17.5 g CaO is mixed with 1 litre sea water.

The reactivity of CaO have also been followed by isothermal calorimetry at 15 °C. The average heat flow curves (mW/g CaO) for three parallel samples at dosage 17.5 g CaO/L are compared for fine and coarse burnt lime at the upper part of Figure 15, while the corresponding cumulative heat curves are plotted at the lower part of Figure 15. The heat flow curves have a bimodal form with a first immediate rapid reaction rate and a secondary increase in the range 30 min (fine CaO) to 70 min (coarse CaO). The secondary reaction was more pronounced for fine CaO. Note that a point on the heat flow curve in Figure 15 (upper part) represent the rate of an exothermal reaction while the slope of an increasing curve represents acceleration. For the cumulative heat in Figure 15 (lower part), a value of about 928 J/g CaO corresponds to a reaction degree of ≈ 80% since the reaction heat for burnt lime in pure water is 1160 J/g CaO, unless there are other exothermal reactions involved like precipitation of brucite. According to the plots in Figure 15, coarse CaO release 928 J/g (80% reaction) after 390 min, while fine CaO develop same amount of energy in 226 min. Note that the reaction rate under adiabatic conditions (developed heat is conserved and temperature rises as in Table 1 and Table 4) will be much faster than under isothermal condition (i.e., constant temperature) as in Figure 15.

**Figure 16:**
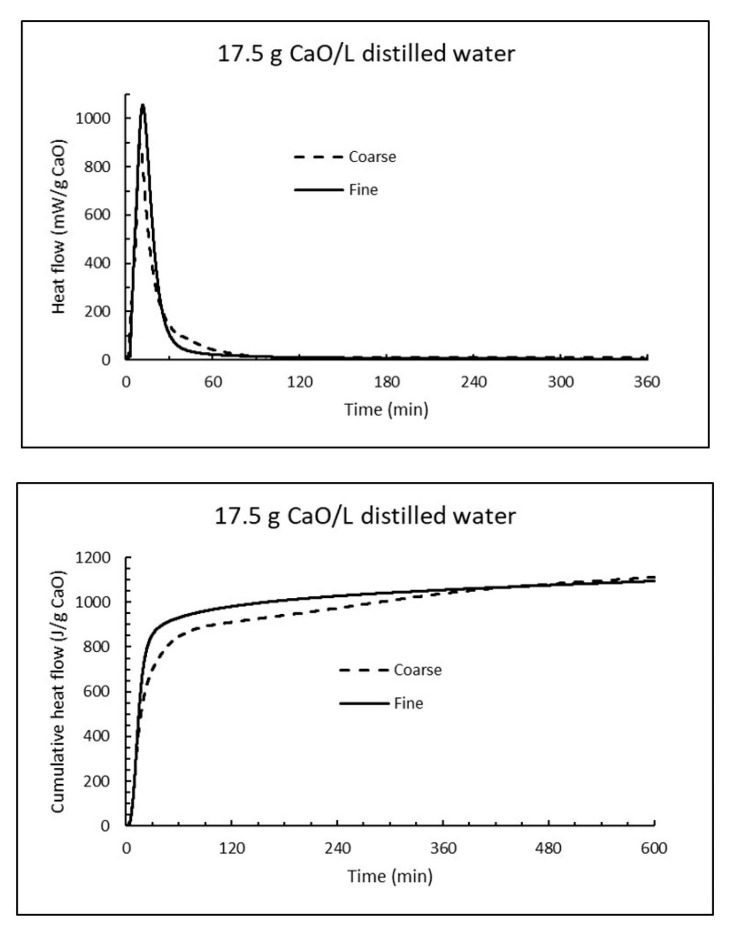
The heat flow (**top**) and cumulative heat (**bottom**) under isothermal conditions at 15 °C when 17.5 g CaO is mixed with 1 litre distilled water.

Analogous isothermal experiments as for sea water were performed for 17.5 g CaO per litre distilled water at 15 °C. The heat flow curves for fine and coarse CaO are plotted at the upper part of Figure 16, while the corresponding cumulative heat curves are shown at the lower part of Figure 16. Evidently the reaction of burnt lime in fresh water is much more intense than in sea water. The time for coarse CaO to reach 928 J/g was 154 min (less than half of that in sea water), while fine CaO used 58 min to reach the same heat evolution compared to 226 min in sea water.

## 4. Discussion

### 4.1. Influence of Burnt Lime on Sea Water Composition as Function of Time

When comparing the evolution of magnesium in Figure 7 with that of calcium in Figure 8 one notice that the concentration of magnesium drops while concentration of calcium increases correspondingly. This is in accordance with Equation (18) postulated from a chemical consideration. From Table 7 it is evident that the concentration of magnesium in sea water at 15 °C has dropped 1263 mg/L after 24 h while the concentration of calcium has increased by 1975 mg/L. Considering the atomic mass of Mg being 24.305 g/mol and that of Ca being 40.078 g/mol, this corresponds to a drop of magnesium of 52 mM and an increase in calcium of 49 mM which is equal in light of measurement uncertainty. The corresponding numbers at 5 °C are a 44 mM drop for magnesium and a 41 mM increase for calcium, again about equal.

The reason why the ion exchange between calcium and magnesium is faster at 15 °C than at 5 °C the first 8 h (see Figure 7 and Figure 8) is that it is likely diffusion controlled and diffusivity is lower at lower temperatures. The temperature dependence of diffusion coefficients [20] can be expressed by

(20) $$D_{(T)\;}{=\;D}_{\left(\mathrm{Tref}\right)}\;\times \;\exp\left\{\frac{\Delta E_{a}}{R}\left(\frac{1}{T_{\mathrm{ref}}}-\;\frac{1}{T}\right)\right\}$$

where ΔE_a_ = activation energy for the reaction (assumed 40 kJ/Mol), R = universal gas constant = 8.3144598 J/K·mol, T_ref_, e.g., 288.15 K (15 °C) versus T = 278.15 K (5 °C) in this case.

The ratio between the diffusion coefficients at 15 and 5 °C is 1.8 using the numbers for Equation (20). The drop in magnesium concentration after 3 h is 948 and 566 mg/L at 15 and 5 °C, respectively, giving a ratio of 1.67. Using the numbers for calcium gives a corresponding ratio of 1.86. This is a strong indication that the Ca^2+^ ↔ Mg^2+^ replacement in the material is diffusion controlled.

The exchange of calcium with magnesium agrees with Nishimuta and Seki [21] studying effects of liming for the improvement of mariculture grounds in a model system. They found that lime transmuted gradually into magnesium hydroxide as a result of the exchange of Ca^2+^ for Mg^2+^, and that lime (CaO) increased pH of the sea water and consequently sulfide and ammonium ion were eluted from the bottom mud. The change in sulphate concentration is somewhat chaotic to start with as seen from Figure 9. It is possible that precipitated magnesium hydroxide blocks some of the surface of the CaO particles in the start, but after a while the concentration decreases successively until it flattens out until 24 h being the latest measuring point. The solubility of gypsum is 1.82 g/L and 1.96 g/L at 5 and 15 °C, respectively, which corresponds to 1020 and 1090 mg SO_4_^2−^/L. The measured concentrations are far higher than that (2662 and 2648 mg/L) after 24 h and shows that the reaction between sulphate anions and calcium hydroxide to gypsum (Equation (19)) is a relatively slow process. The decay in in the sulphate concentration in the first 24 h is not more than 4–6%.

The carbonate concentration drops and levels out on a plateau when 1% fine CaO is added sea water (Figure 10). The value of the plateau cannot be compared to the solubility of calcium carbonate since it is not an absolute value, but a relative value measured as the area under a curve.

The change in pH as function of time when 1% fine CaO is added sea water in Figure 11 shows that pH increases rapidly (within 1 min) from the pH of sea water of 7.9 at 5 °C and 7.7 at 15 °C to 10.6 and 10.1, respectively. The low measured pH relative to calcium hydroxide is because the CaO dosage was so small that Mg^2+^ was not depleted as seen from Figure 7. Hence, the pH is regulated by precipitated magnesium hydroxide. This will also be the case at open sea where magnesium is in excess.

The dissolution of magnesium hydroxide can be written as;

Mg(OH)_2_ (s) = Mg^2+^ + 2 OH^−^ with K_sp_ = [Mg^2+^] × [OH^−^]^2^ = 5.61 × 10^−12^ (21)

Dispersing magnesium hydroxide in fresh water means that [OH^−^] = 2 [Mg^2+^] and [OH^−^] = $\sqrt[{3}]{2K_{\mathrm{sp}}}$ = 2.2 × 10^−4^ corresponding to pH = 10.4. From Figure 7 it can be seen that after 8 h at 15 °C [Mg^2+^] = 149 g/L = 6.13 mM and [OH^−^] = $\sqrt{K_{\mathrm{sp}}/[{\mathrm{Mg}}^{2+}]}$ = 29.8 × 10^−6^ corresponding to pH = 9.5 while it is measured 9.8. This is just serving as an illustration and it is without taking into temperature effect or complex ions etc. At 5 °C it seems like situation is far from equilibrium. At open sea where magnesium is in surplus and concentration is 53.6 mM (Table 4), the pH at equilibrium would have been reduced to 9.0 at 25 °C and lower than that at lower temperatures since solubility of magnesium hydroxide decreases with decreasing temperature (0.009 g/L at 18 °C and 0.04 g/L at 100 °C [19]) unlike calcium hydroxide (Equation (3)).

### 4.2. Change of Solids Composition as Function of Time for CaO in Contact with Sea Water

The results in Table 7 reveal that calcium hydroxide is formed immediately (first minute) and in a relatively large amount when 1% fine CaO is mixed with sea water at 5 °C, since only water is needed, and it is in large excess. Magnesium hydroxide and calcium carbonate are also formed relatively quickly even though the amounts of reactants are limited in this “closed system” and is controlled by amount of CaO relative to sea water and the content of magnesium and carbonate in the sea water. In practical applications the system is “open”, and the supply of reactants are unlimited. The availability of reactants then relies on a combination of current/convection and diffusion of species.

**Figure 17:**
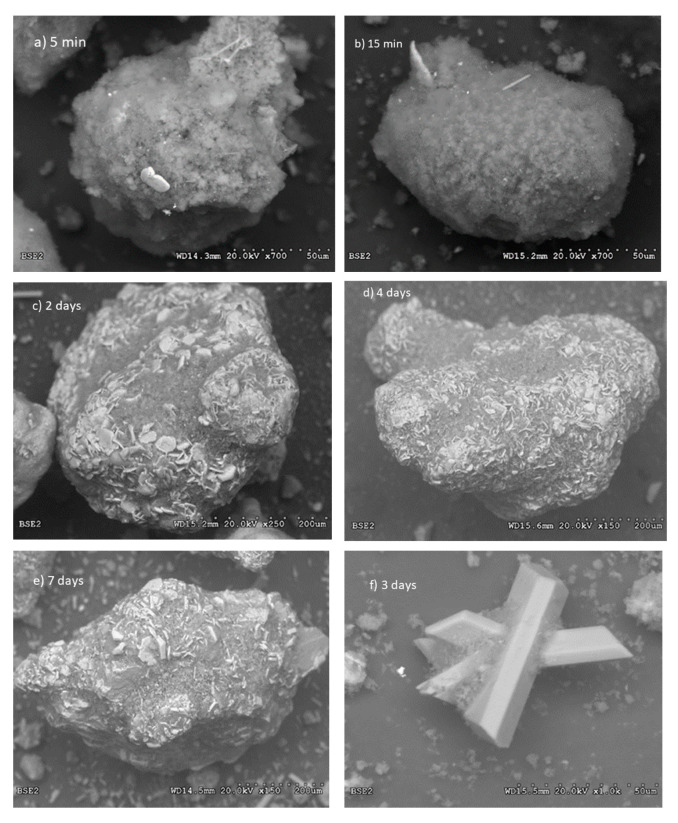
A selection of images from the SEM investigation as a function of time for lime particle; (**a**) 5 min, (**b**) 15 min, (**c**) 2 days, (**d**) 4 days and (**e**) 7 days, as well as (**f**) after 3 days showing gypsum crystals.

It is noticeable from Table 7 that the formation of gypsum occurs slower than the other compounds and appears first after 5 h. The presence at this time is confirmed by XRD and can be seen as prismatic crystals by SEM in Figure 17. Another feature disclosed by SEM is that a grey mass is precipitated early on the particle. This is likely to be magnesium hydroxide and may be the reason why burnt lime hydrates slower in sea water than in fresh water as seen from the calorimetry curves in Figure 15 and Figure 16. Later calcium hydroxides crystallizes to hexagonal plates in line with its crystal structure that penetrates through the precipitated mass of magnesium hydroxide. Examples of this are shown in Figure 17.

Images (a) and (b) in Figure 17 depicts seemingly amorphous precipitations (likely magnesium hydroxide) on the lime particles after 5 and 15 min, respectively, while image (c) after two days shows that calcium hydroxide has crystallized to small hexagonal plates penetrating the precipitated grey mass on the particle surface. Magnesium hydroxide will appear darker grey than calcium hydroxide since it is composed of lighter elements, but the physical density may also affect the grey levels. Hexagonal crystals of calcium hydroxide also appear on the particle surface after 4 d in image (d) and image (e) shows various crystal shapes on the particle surface after seven days. Image (f) focuses on well-crystallized prisms of gypsum at an age of three days. Note that the magnification indicated by the dotted line at the bottom of the images is 50 μm for image (f) and 200 μm for the other images.

### 4.3. Burnt Lime Added to Sea Water in an “Open System”

Very few trials have been performed to measure pH when burnt lime has been dosed directly to open sea. In such an “open system” will the calcium hydroxide formed during hydration have access to magnesium and other components for neutralization, and pH will be a question of time and distance from individual burnt lime particles.

The following test was performed in the river Murray in Canada as described in the (Aquaculture and Rural Development) DFARD Tech Report #253 [22]: The sea water just outside the river mouth had pH 8.1, while the pH in a trough of slaked lime (i.e., calcium hydroxide) was measured to 12.6. When the slaked lime was thrown into the sea, the pH within the cloud of particles was up to 9. The sea water about 10 m from the trough, but directly above the treatment zone, had pH in the range 8.2–8.3. The pH values dropped rather rapidly back to the natural background level of 8.1 close to the treatment zone.

**Figure 18:**
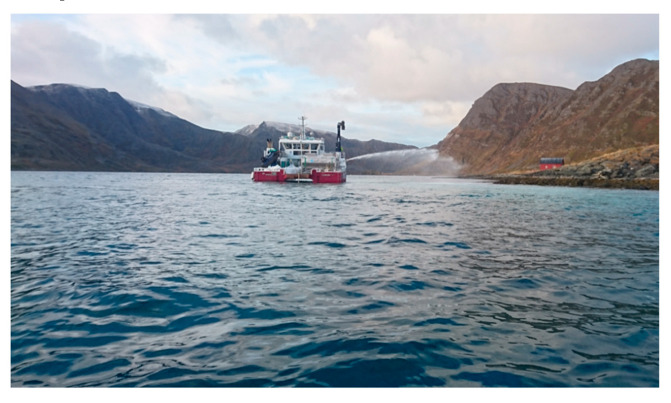
Photo of the boat spraying lime suspension of coarse CaO over the sea.

**Figure 19:**
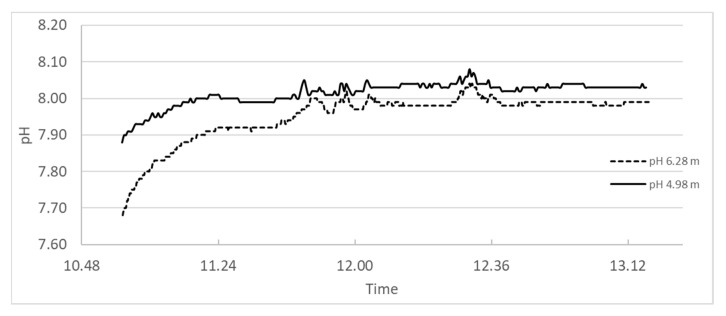
pH measured in open sea in the area where a suspension of coarse CaO was sprayed.

In 2017 [23] and experiment was performed in the Slettnes fjord near Hammerfest in northern Norway, where a suspension of burnt lime was spread over the sea where pH-meters were mounted at 4.98 and 6.28 m depths. A photo of the boat spraying lime suspension is shown in Figure 18, while the pH logs for the whole period (2 h and 24 min) are plotted in Figure 19.

The measurement at depth 4.98 m starts at 7.8 and climbs to pH 8.0 within 20 min after spraying starts and the highest pH measured within the period of 2 h and 24 min was 8. 1. The measurement at depth 6.28 m started with pH 7.2 and climbed to 7.9 within 20 min and stabilized at 8.0 within the measurement period and with 8.0 as the highest value read. It is worth noting that all values measured are within the range to be expected in natural sea water.

The measured pH at open sea is in line with the pH estimation <9 using Equation (21) and sea water concentration of Mg^2+^ at temperatures <20 °C.

**Figure 20:**
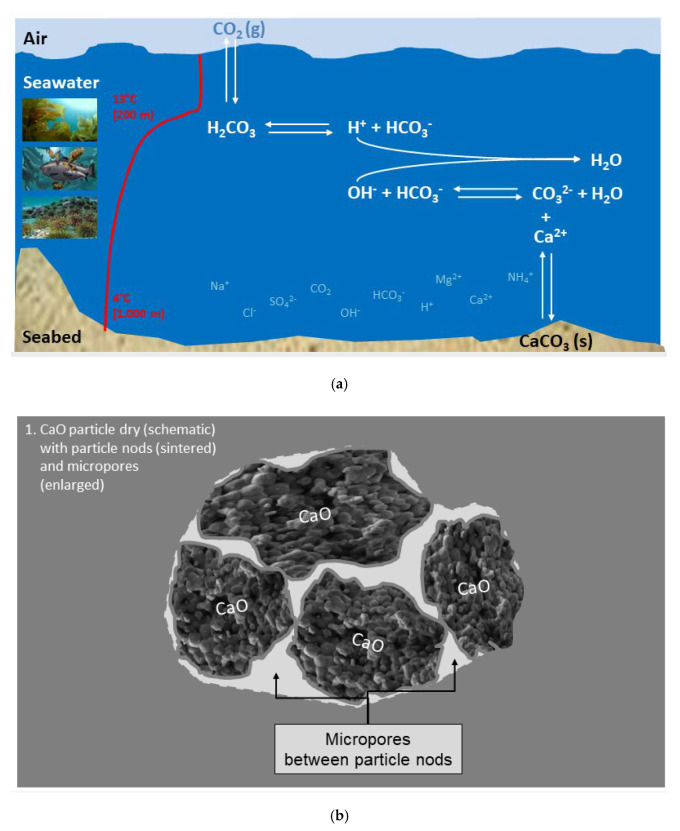

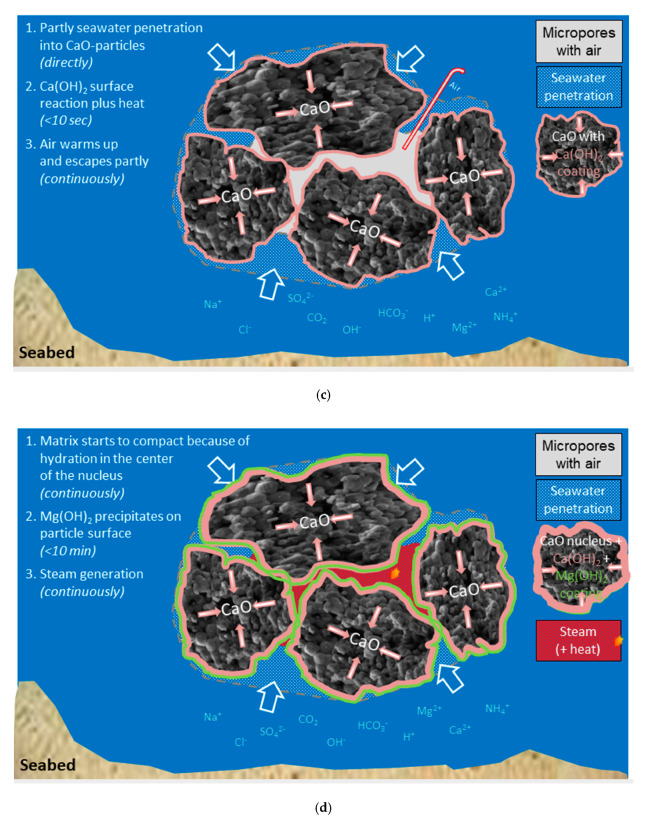

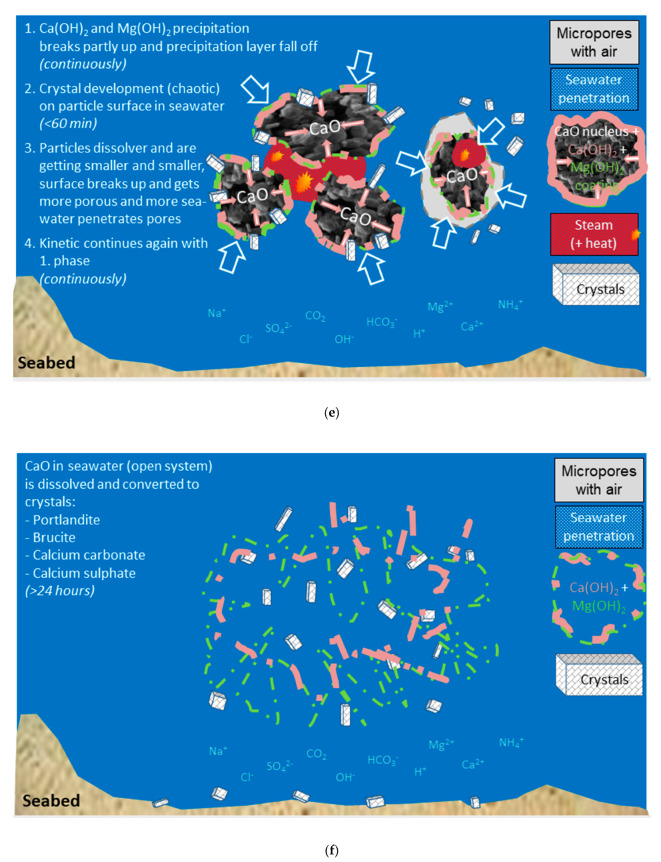
(**a**) Sketch of the «open system» sea water with temperature profile and different ions; (**b**) Sketch of an agglomerate of burnt lime particles; (**c**) **Phase 1**: Immediate reaction between burnt lime and sea water—a surface layer of calcium hydroxide is formed; (**d**) **Phase 2**: The surface reaction forming calcium hydroxide continues and magnesium hydroxide is precipitated; (**e**) **Phase 3**: Structural compression and formation of crystals; (**f**) **Phase 4**: All burnt lime particles are completely reacted and transformed to other solids.

Based on the experimental data retrieved, observations like those depicted in Figure 17 and theoretical evaluation, the reaction progress when burnt lime is mixed into the sea can be described as sketched in Figure 20a–f.

## 5. Conclusions

The reaction of burnt lime in sea water has been discussed and compared to fresh water.

A particle of burnt lime (CaO) added to sea water will immediately react to form calcium hydroxide (called slaking) and more than 90% of the lime would have reacted within 5 h in a “closed system”. This process is expected to be even faster in an “open system” such as the open sea or a fjord. The slaking process is extremely exothermic and will be able to increase the temperature of the surroundings of the particles. However, a remarkable or persistent increase of local sea water temperature would be extremely unlikely due to the low doses applied and the large water reservoir.

The reaction of burnt lime in sea water is slower than in fresh water. This is probably due to the large concentration of magnesium in sea water that provokes a rapid precipitation of magnesium hydroxide onto the surface of the particle and hinders the progress of the hydration reaction of CaO.

The slaking process will increase pH locally from the natural level of about 8 to a theoretical maximum of 12.5 near the surface of the particle on a micro level. In an “open system” the pH will be within the variation in natural sea water (7.8–8.5) a few cm away from the particle, as shown in practical field experiments.

In parallel to the slaking process, dissolved calcium hydroxide will react with magnesium ions in sea water and precipitate less soluble magnesium hydroxide. The equilibrium pH of magnesium hydroxide in fresh water is 10.5, but in equilibrium with the magnesium concentration in sea water it is calculated to < 9 as observed.

All carbonate, hydrogen carbonate and dissolved CO_2_ in the vicinity of formed calcium hydroxide will be consumed and yield calcium carbonate. Same happens with sulphate, that will react and form gypsum, but somewhat slower than the magnesium and carbonate reactions. It is likely however that the magnesium reaction will dominate in an “open system”.

The product formed at equilibrium after dosing burnt lime in the sea will be a mixture of magnesium hydroxide (that possibly will be slowly converted to magnesium carbonate over time), calcium carbonate and gypsum (i.e., calcium sulphate dihydrate). All calcium oxide and hydroxide will be consumed and no traces of them will remain at equilibrium. How long time that will take depends on the thinning rate probably being a function of the particle size, the temperature and the water renewal due to natural currents. Correspondingly will all dissolved calcium be diluted and eventually reach the natural background level of sea water. According to the data retrieved in this study, the reaction will be complete within a few days or maximum a week. It is worth noting that all of the final products magnesium carbonate, calcium carbonate and gypsum occur naturally and can be regarded harmless for the environment.

## Figures and Tables

**Table 1:** Temperature increase (ΔT; °C) in water as function of dosage of burnt lime and its hydration fraction as calculated from Equations (1) and (2).

Dosage →/Hydration Fraction ↓	200 g/L	150 g/L	100 g/L	50 g/L
0.2	11	8	6	3
0.4	22	17	11	6
0.6	33	25	17	8
0.8	44	33	22	11
1.0	56	42	28	14

**Table 2:** Typical composition of Atlantic sea water.

Species	M_w_ (g/mol)	Mass (%)	M (mol/L)
Na^+^	22.990	1.08	0.4825
Cl^−^	35.453	1.94	0.5620
Mg^2+^	24.305	0.13	0.0549
SO_4_^2−^	96.063	0.27	0.0289
Ca^2+^	40.078	0.04	0.0103
K^+^	39.098	0.04	0.0105
Br^−^	79.904	0.0067	0.0009
HCO_3_^−^	61.017	0.005	0.0008
CO_3_^2−^	60.009	0.001	0.0002

**Table 3:** Potential compounds that can form by interaction of CaO with sea water.

Compounds	M_w_ (g/mol)	Solubility (g/L)	Concentration (mM)
Ca(OH)_2_	74.09	1.77	23.89
CaCO_3_^a^	100.09	0.014	0.14
Mg(OH)_2_	58.32	0.009	0.15
MgCO_3_^b^	84.31	0.106	1.26
CaSO_4_·2H_2_O	172.17	2.5	14.52
MgCO_3_·Mg(OH)_2_·3H_2_O ^c^	196.68	-	-
3MgCO_3_·Mg(OH)_2_·3H_2_O ^b^	365.31	0.4	1.09
MgCO_3_·5H_2_O ^b^	174.39	1.8	10.32
MgCO_3_·3H_2_O ^b^	138.36	1.6	11.56

^a^ Can be found as the polymorphs calcite, aragonite and vaterite which can be differentiated by XRD. ^b^ Not expected to form as the molar solubility is greater than for Mg(OH)_2_. ^c^ Solubility not known, but the molar solubility is expected to be greater than for Mg(OH)_2_ and it is not likely to form.

**Table 4:** Properties of fine and coarse burnt lime.

Property	Method	Fine CaO	Coarse CaO
Specific surface (m^2^/g)	BET	1.53	1.12
Active CaO (%)	ASTM C25-11 E1	94	91
CO_2_ residue (%)	ASTM C25-11	1.6	2.7
Reactivity in fresh water	EN 459-2	-	-
Time to reach 60 °C (s)		100	250
Semi-adiabatic ΔT (°C)		75	75
Reactivity in sea water	EN 459-2	-	-
Time to reach 60 °C (s)		300	500
Semi-adiabatic ΔT (°C)		70	70

**Table 5:** Major ion composition and pH of the sea water used.

Species	M_w_ (g/mol)	ppm	M (mol/L)
Na^+^	22.990	10,981.1	0.4776
Cl^−^	35.453	18,553.6	0.5233
Mg^2+^	24.305	1301.6	0.0536
SO_4_^2−^	96.063	2835.3	0.0295
Ca^2+^	40.078	399.0	0.0100
K^+^	39.098	394.0	0.0101
Br^−^	79.904	0.05	0.0000
F^−^	18.998	1.25	0.0001
pH	-	7.88	-

**Table 6:** Compositional change of sea water at 5 and 15 °C the first 24 h after adding 1% *w*/*v* fine CaO.

Species	Temperature 5 °C		Temperature 15 °C	
	Time 0 h	Time 24 h	Time 0 h	Time 24 h
Magnesium, Mg^2+^	1302 mg/L	236 mg/L	1265 mg/L	2.1 mg/L
Calcium, Ca^2+^	399 mg/L	2056 mg/L	386 mg/L	2361 mg/L
Sulphate, SO_4_^2−^	2835 mg/L	2662 mg/L	2764 mg/L	2648 mg/L
Carbonate, CO_3_^2−^	0.046 μS·min	0.035 μS·min	0.045 μS·min	0.061 μS·min

**Table 7:** Bulk composition (%) of solids from reaction of 1% fine CaO with sea water versus time at 5 °C for the first 24 h.

Time	1 min	5 min	15 min	30 min	1 h	2 h	5 h	8 h	24 h
Time (min)	1	5	15	30	60	120	300	480	1440
Gypsum	0.0	0.0	0.0	0.0	0.7	0.0	8.5	4.3	0.4
Mg(OH)_2_	9.0	17.7	7.1	14.8	16.1	5.0	17.0	15.2	12.7
Ca(OH)_2_	50.4	49.0	55.4	66.2	61.3	70.3	62.7	69.9	76.4
CaCO_3_	7.7	8.2	5.7	8.7	7.0	4.9	6.4	6.0	6.3
CaO	30.1	20.9	30.4	7.6	12.3	18.8	4.2	3.2	2.9
Sum	97.3	95.8	98.6	97.3	97.5	99.0	98.8	98.6	98.7

**Table 8:** Bulk composition (%) of solids from reaction of 1% fine CaO with sea water versus time at 5 °C for 1–7 days.

Time	1 d	2 d	3 d	4 d	5 d	6 d	7 d
Gypsum	0.4	3.1	8.4	5.3	5.8	9.5	8.2
Mg(OH)_2_	12.7	16.4	17.6	12.3	21.3	16.0	14.7
Ca(OH)_2_	76.4	72.6	64.6	73.4	62.2	68.8	70.0
CaCO_3_	6.3	5.8	8.6	8.5	9.7	5.3	6.4
CaO	2.9	1.0	0.0	0.0	0.0	0.0	0.0
Sum	98.7	98.8	99.2	99.4	99.0	99.6	99.3
